# Successful Treatment of a Nonunion Fracture of the Femur With Extracorporeal Shockwave Therapy: An Evidence-Based Case Report

**DOI:** 10.7759/cureus.84138

**Published:** 2025-05-14

**Authors:** Theodore Tandiono, Jessica Amelinda Mintarjo, Nunung Nugroho

**Affiliations:** 1 Department of Physical Medicine and Rehabilitation, Primasatya Husada Citra Hospital, Surabaya, IDN

**Keywords:** eswt, evidence-based case report, non-invasive treatment, nonunion femur fracture, nonunion fracture

## Abstract

Nonunion fractures, especially of the femur, pose a considerable difficulty in orthopedic management due to their propensity to result in enduring physical disability and psychological complications. Conventional surgical procedures, although efficacious, may entail difficulties and have a reduced success rate for atrophic nonunions. Extracorporeal shockwave therapy (ESWT) has arisen as a viable non-invasive option, facilitating bone regeneration via many biological mechanisms. This case report describes the management of a 40-year-old male patient with a distal-third open femoral fracture resulting from a motor vehicle accident. Despite receiving open reduction internal fixation and an extensive rehabilitation regimen, the patient had little callus development after six months. ESWT was commenced once weekly for 23 weeks, combined with ultrasonic low-intensity pulsed ultrasound (LIPUS) therapy and leg extension exercises. The patient had full callus formation on the femur and made a full recovery. The comprehensive literature evaluation demonstrates that ESWT can achieve a healing rate of around 73% for nonunion femoral fractures. The therapy's effectiveness is due to its capacity to induce osteogenic responses, promote osteoblast development, and suppress osteoclast production. This non-invasive method diminishes the necessity for supplementary surgical interventions and related complications. Additional research is required to develop standardized methods and refine therapy parameters, guaranteeing consistent and dependable results across various patient groups.

## Introduction

One of the most difficult orthopedic disorders to treat is pseudoarthrosis, sometimes called nonunion. The incidence, which ranges from 5% to 10% and might reach 50%, differs substantially based on the kind of fracture, the location in the body, and whether the fracture is open or closed. Nevertheless, it is anticipated that the frequency will rise due to the better survival rates observed in individuals with polytrauma [1,2]. Patients may experience long-term physical impairment and mental health issues due to nonunions, which can place a significant financial strain on society [3].

Approximately 80% of patients achieve satisfactory to outstanding ultimate restoration of mechanical axis alignment and appropriate length after undergoing one of the several surgical procedures used to address nonunion [4]. But these findings encompassed all forms of nonunions; the success percentage would be much lower for atrophic nonunions. Additionally, there is a significant decrease in the healing rate when several procedures are necessary. So, to improve nonunion healing, bone regeneration techniques have been used. The most effective method at the moment is autologous bone grafting, but this material is in short supply, and its healing capabilities are uncertain [5]. In addition, it is associated with harvesting-related morbidities and necessitates an extra surgical site [6].

While nonunion surgery is often an essential component of treating displaced nonunions, it might backfire if the fixation is too tight or the surgeon fails to achieve proper alignment, among other possible complications.

An alternative to surgical procedures for treating nonunions is stem cell therapy, which includes procedures such as percutaneous bone marrow aspirate concentrate (BMAC) transplantation [7]. For patients with delayed healing or definitive nonunions, another conservative therapeutic option is extracorporeal shockwave therapy (ESWT) [8-10]. Several experimental investigations found that shockwaves (SWs) induce transforming growth factor β1 (TGF-β1) and vascular endothelial growth factor (VEGF) induction, which in turn promote the development and differentiation of mesenchymal stem cells (MSCs) toward osteo-progenitors [11,12]. Yet another research found that ESWT stimulates MSC osteogenic differentiation via P2x7 receptors by inducing ATP release [13]. Although there is still no agreement about ESWT's capacity to control SDF-1 expression, other research has shown that SWs may also enhance the expression of other important factors, such as SDF-1 [14-18]. The repopulation and homing of MSCs in bone marrow are facilitated by SDF-1, which was finally identified [19].

In addition, sufficient blood flow is necessary for stem cell migration to take place, and SWs promote neovascularization and bone healing [18-20]. Researchers found that SWs increased the treated trabecular bone's thickness and volume in animal tests [21,22]. In conclusion, ESWT promotes bone repair without intrusive procedures.

According to the literature, the success rate of ESWT in treating long bone fracture nonunions varies from 54% to 98%. This range is influenced by factors such as the location of the injury, the kind of nonunion, and the amount of time that has passed from the accident before treatment [23,24]. The radiological aspect is the standard for classifying nonunions. Excessive mobility and a thick bone callus are hallmarks of hypertrophic nonunion, which may have biological healing potentials in theory. Oligotrophic nonunion is characterized by insufficient callus development, elevated mobility, and compromised biology. Because of decreased vascularity and a lack of biological healing capacity, callus development does not occur in an atrophic nonunion [25].

When it comes to long-bone hypertrophic nonunions, ESWT has the ability to heal just as well as surgery in certain instances [26]. On the other hand, ESWT has been found to have significantly less success with atrophic nonunions [27].

The research reports inconsistent outcomes, and there is no agreement on the case-specific parameters to use during ESWT treatments, despite the fact that it can be a good alternative to surgery. This evidence-based case report presented a case of nonunion fracture that was treated with ESWT and discussed evidence supporting this treatment that was given to our patient.

## Case presentation

**Figure 1:**
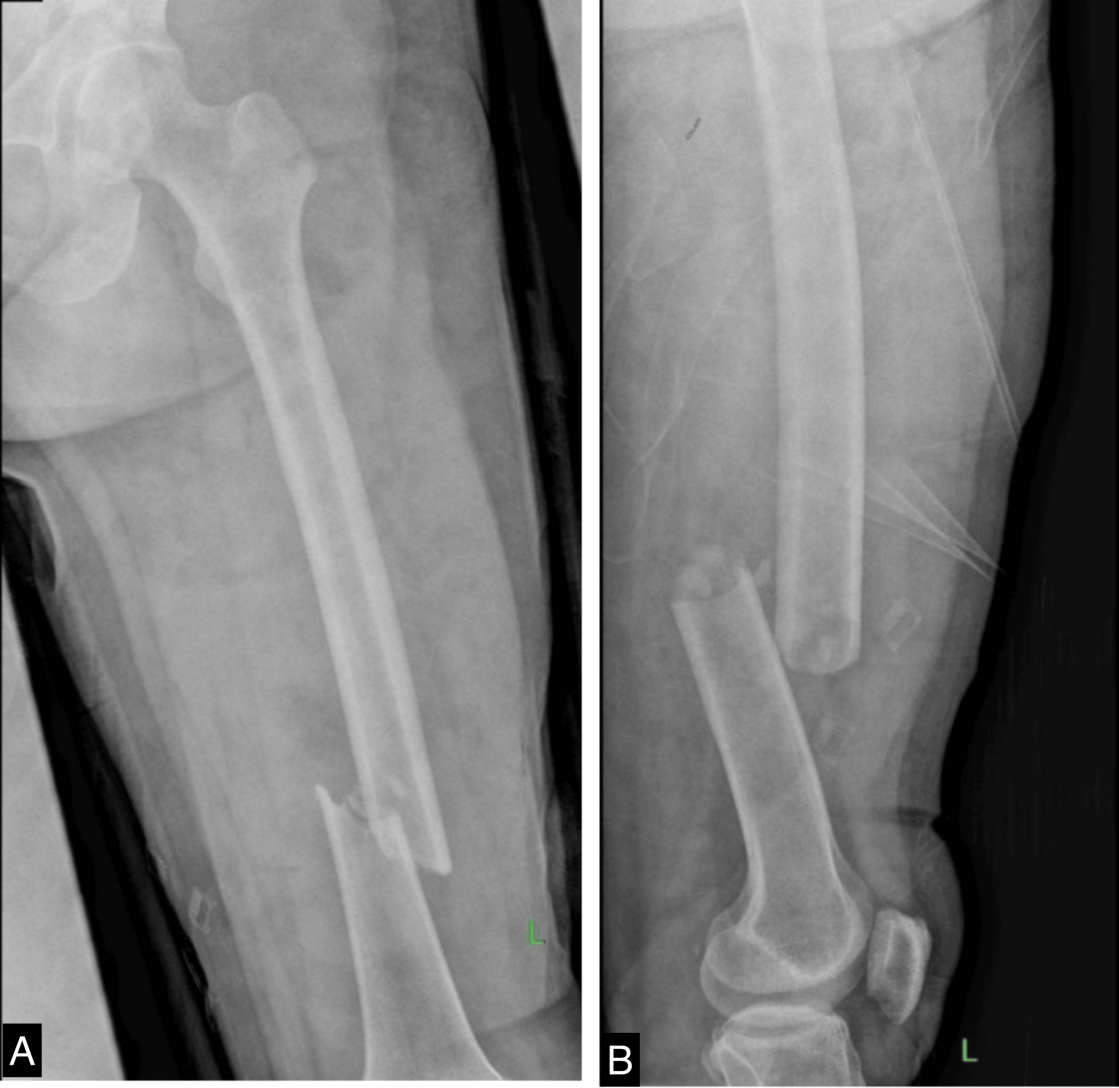
X-ray of the left femur at the ER shows a distal-third open fracture of the left femur. A. anteroposterior view; B. lateral view

**Figure 2:**
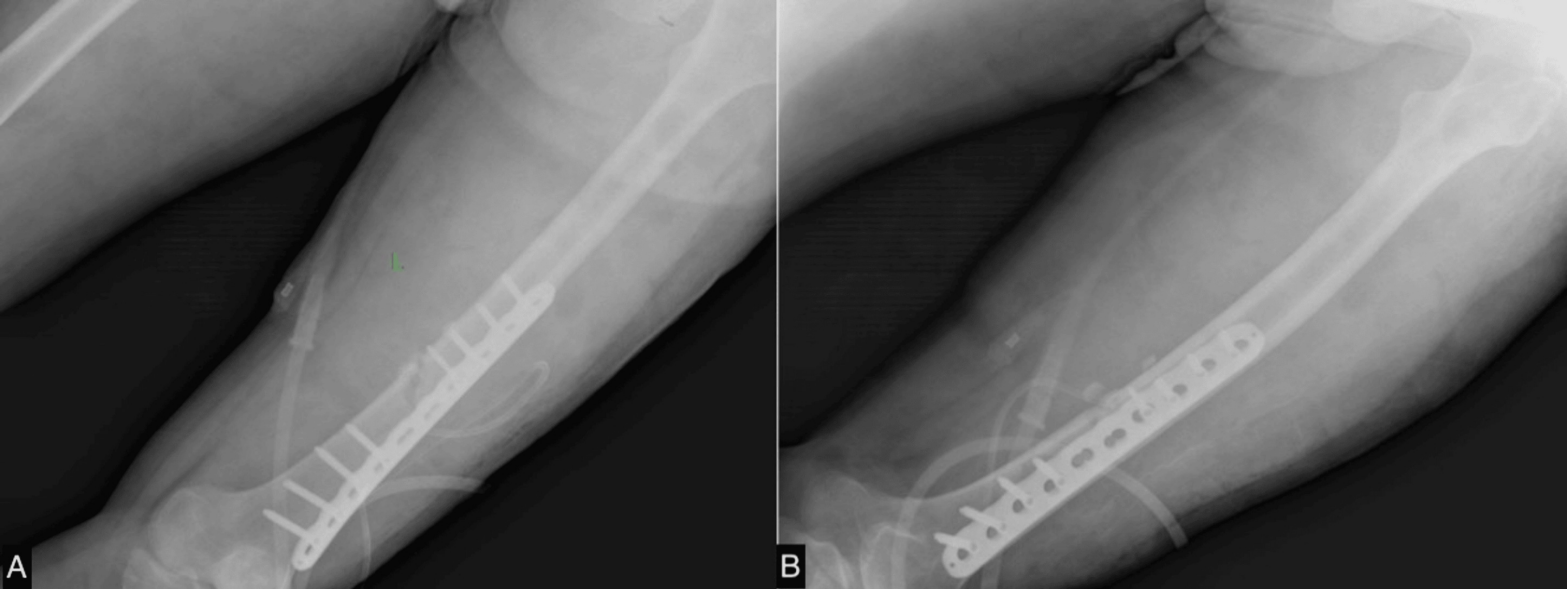
X-ray of the left femur obtained after open reduction internal fixation A. anteroposterior view; B. lateral view

A 40-year-old male patient sustained a distal-third open fracture of the femur in his left thigh following a motor vehicle collision, as it is shown in Figure 1. Fortunately, he did not sustain any head injuries during the incident. An open reduction internal fixation and debridement procedure was performed to address the fracture. Postoperative X-rays (Figure 2) revealed no signs of surgical complications. Subsequently, the patient was referred to a physiatrist to commence the rehabilitation program. The patient weighed 66 kg and was 166 cm tall with a normal body mass index (BMI) of 24 kg/m². The patient had no past medical or drug history. To facilitate bone healing, the patient was prescribed two supplements, namely, calcitriol 0.5 mcg and a supplement containing ossein hydroxyapatite compound (OHC) 800 mg. Both supplements were to be consumed daily.

**Figure 3:**
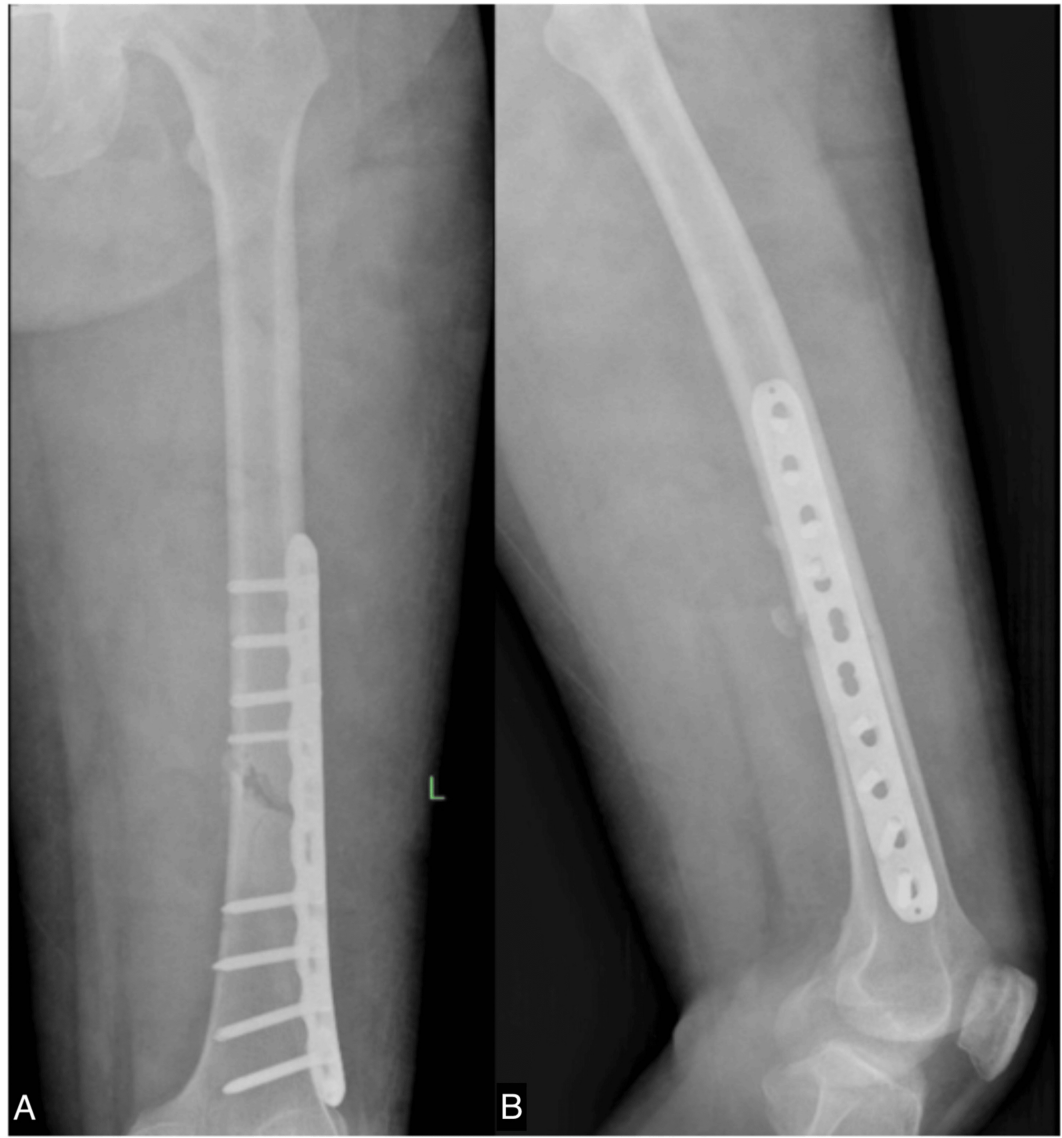
X-ray of the left femur obtained two months postoperatively A. anteroposterior view; B. lateral view

The initial phase of rehabilitation, after evaluating the X-ray as shown in Figure 3, spanning the first two months, involved passive training on THERA-Trainer (Medizintechnik GmbH, Hochdorf, Germany) electrical stimulation of the vastus medialis and lateralis muscles, ankle pumping, and quadriceps strengthening exercises. These exercises were aimed at strengthening lower extremity muscles and promoting blood flow to reduce swelling and facilitate healing. Subsequently, the patient underwent ultrasound diathermy (USD) to induce bone healing. Electrical stimulation of the medial and lateral vastus muscles, leg extension exercises with a maximal range of motion (ROM) of 0-70 degrees, and continued use of the THERA-Trainer for lower extremity exercises were also incorporated into the rehabilitation regimen. Over the next three months, the patient experienced significant improvements in his leg function. His ROM increased, and he was able to walk without the assistance of bilateral crutches. However, X-rays performed three months after surgery revealed no evidence of callus formation. The rehabilitation program continued with ultrasonic low-intensity pulsed ultrasound (LIPUS), leg extension exercises, THERA-Trainer use for lower extremity exercises, and electrical stimulation for thigh muscle strengthening. These interventions were implemented for another three months, during which time X-rays showed minimal callus formation. The patient was able to walk without the use of assistive devices. However, he experienced occasional pain during prolonged walking and stair climbing. 

**Figure 4:**
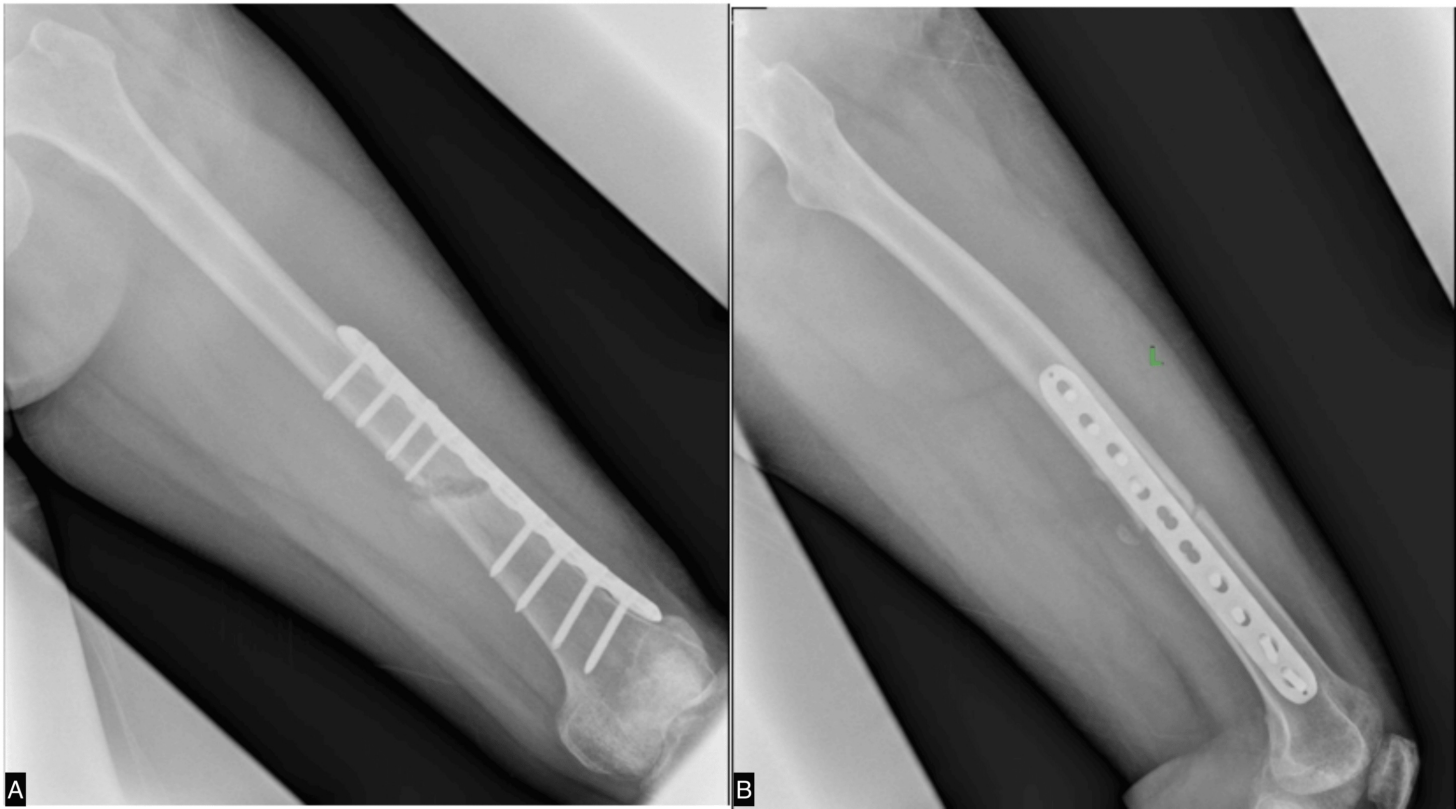
X-ray of the left femur obtained six months postoperatively shows barely any callus formation. A. anteroposterior view; B. lateral view

**Figure 5:**
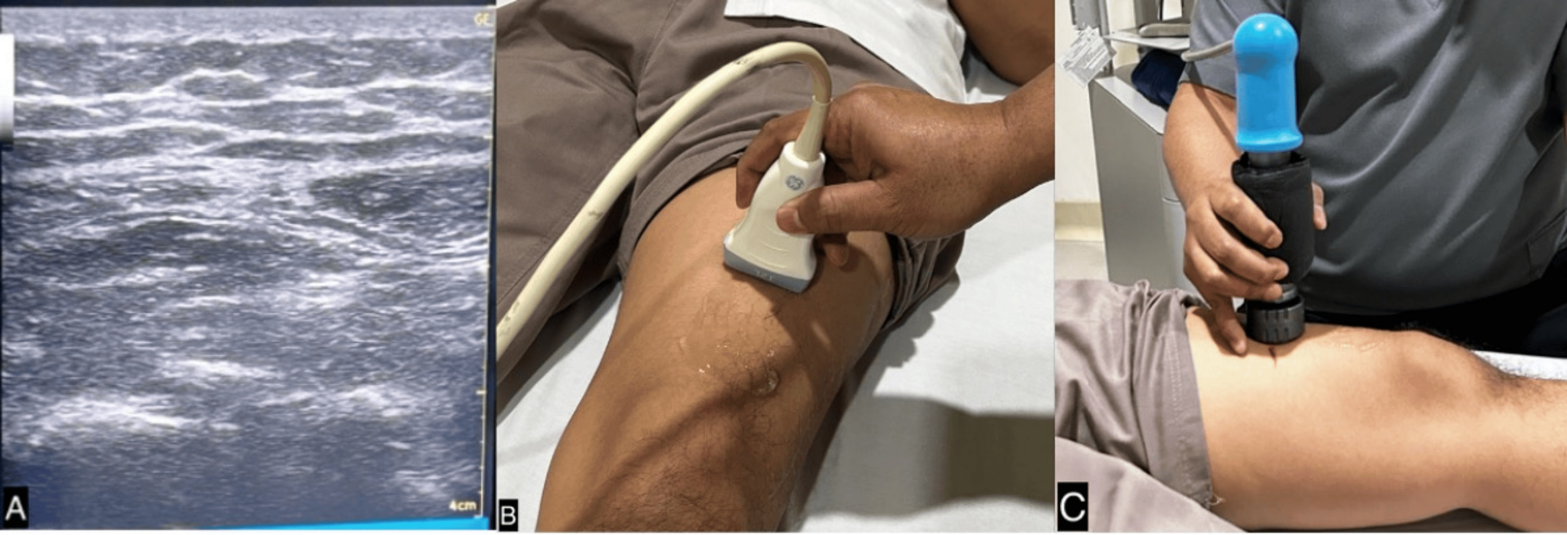
(A) Ultrasound sonography (USG) of the left femur, right on the nonunion fracture; (B) USG probe location of the patient's left femur; (C) Treatment of the patient using ESWT (EMS SwissDolorClast Master with a 15 mm head size and EvoBlue handpiece) settled on one point at the fracture site. ESWT: extracorporeal shockwave therapy

**Figure 6:**
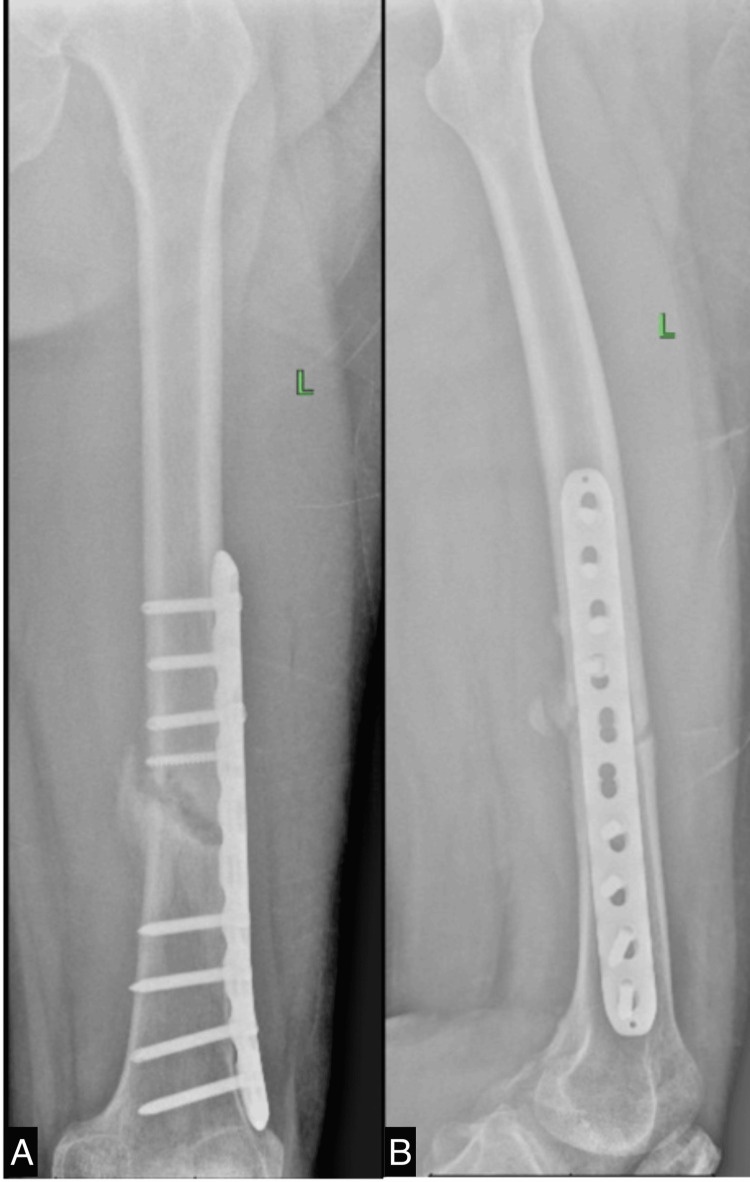
X-ray of the left femur obtained seven months postoperatively and after four sessions of ESWT shows callus formation. A. anteroposterior view; B. lateral view ESWT: extracorporeal shockwave therapy

**Figure 7:**
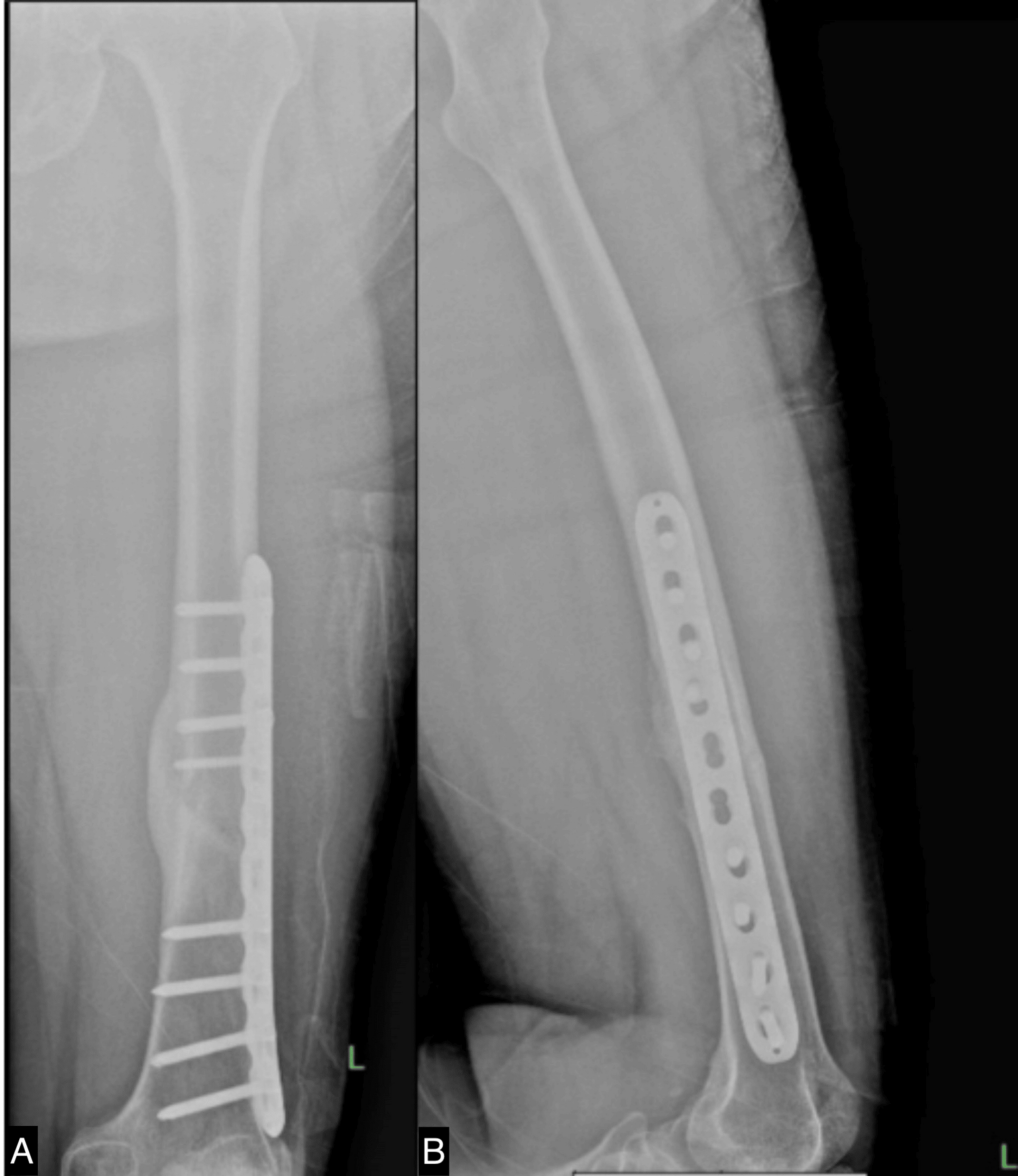
X-ray of the left femur obtained almost six months postoperatively and after 23 sessions of ESWT shows full callus formation. A. anteroposterior view; B. lateral view ESWT: extracorporeal shockwave therapy

**Figure 8:**
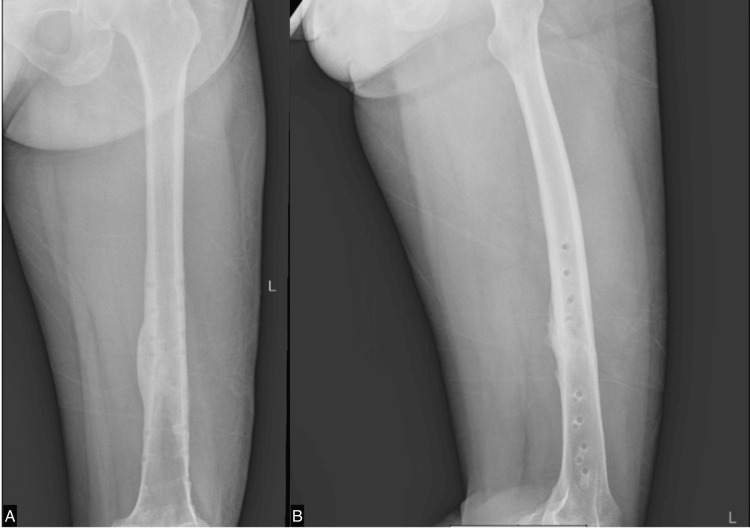
X-ray of the left femur obtained after implant removal A. anteroposterior view; B. lateral view

After six months of rehabilitation without any evidence of osteogenesis, as shown in Figure 4, we commenced ESWT once weekly. Initially, the site of the nonunion fracture was localized using ultrasound guidance. A marker was then placed at the specific fracture location. We employed the EMS Swiss Dolorclast Master (EMS, Nyon, Switzerland) with a 15 mm head size and an EvoBlue handpiece (EMS), all shown in Figure 5. A single point was positioned at the fracture site, applying a pressure of 2.0 bar, delivering 5000 impulses, and maintaining a frequency of 10 Hz for each treatment. Following four sessions, X-rays were conducted to assess the progress shown in Figure 6. Although the callus formation was still minimal, it demonstrated a significant improvement compared to the initial six months after surgery without ESWT. Subsequently, ESWT was combined with ultrasonic LIPUS therapy to stimulate bone healing and leg extension exercises to strengthen the thigh muscles. After 23 weeks of ESWT (given once every week), X-rays revealed complete callus, as seen in Figure 7. The rehabilitation continued to focus on strengthening the thigh muscles before the implant removal surgery. Following the surgery, the patient achieved a full recovery, as shown in Figure 8.

## Discussion

Search strategy

**Figure 9:**
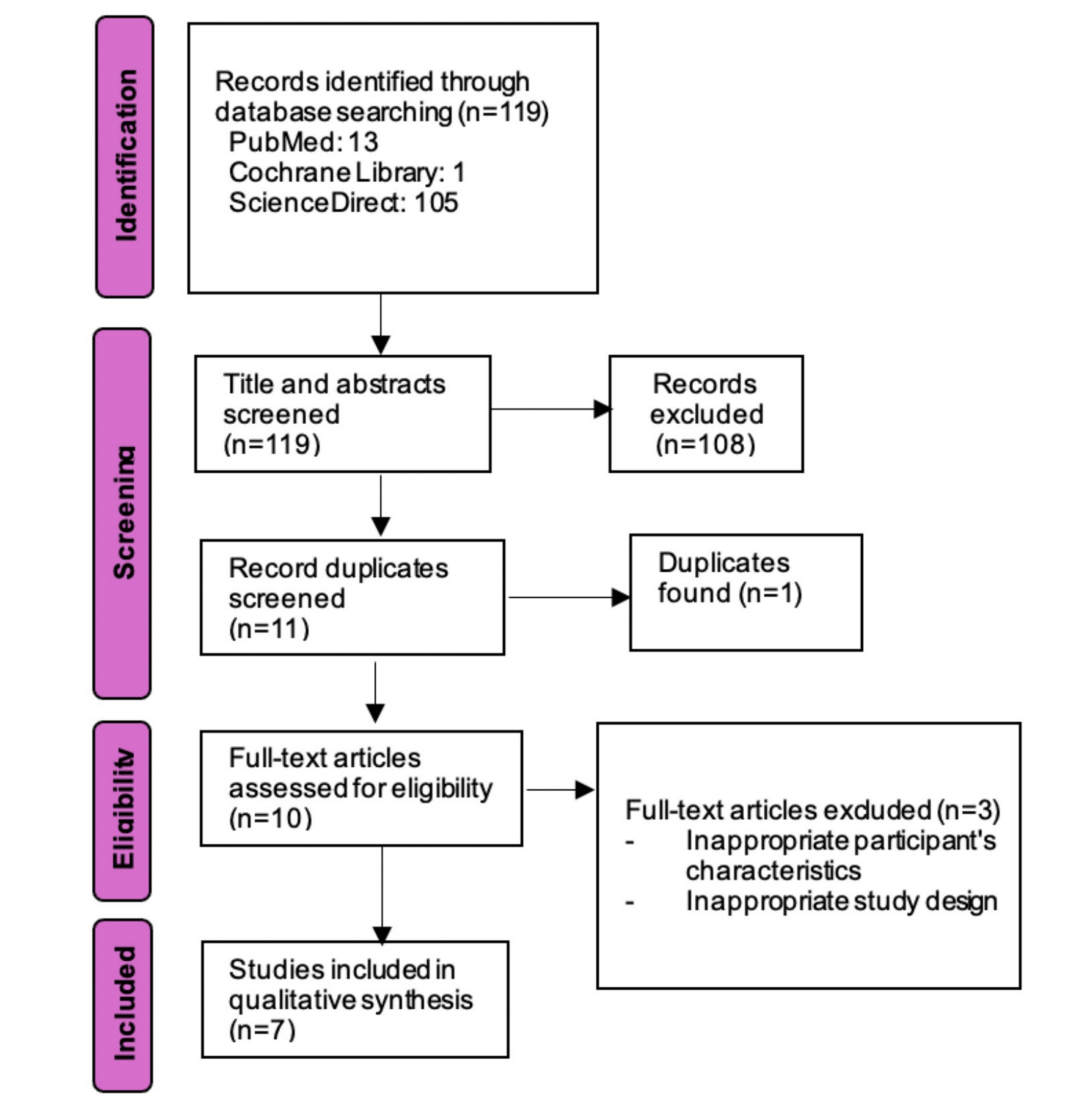
A PRISMA flowchart outlining the literature search strategy for this systemetic review PRISMA: Preferred Reporting Items for Systematic Reviews and Meta-Analyses

This evidence-based case report adhered to the 2020 standards of the Preferred Reporting Items for Systematic Reviews and Meta-Analysis (PRISMA). We conducted a comprehensive literature review utilizing PubMed, Google Scholar, Cochrane Library, and ScienceDirect. The preset keyword utilized throughout the literature search was "(Nonunion fracture) AND (Femur) AND (Extracorporeal Shock Wave Therapy OR ESWT)." In this step, articles with relevant titles and abstracts were selected for further qualitative analysis and comprehensive assessment of the complete text. The articles included in this review were required to be written in English, published between 1995 and 2025, and possess accessible full texts. The requirements of the research search technique are illustrated in Figure 9.

Inclusion and exclusion criteria

The main aim of the study selection criteria was to uncover studies that provided extensive information on the use of ESWT in treating nonunion femur fractures. To ensure a comprehensive analysis, only papers that met these criteria were considered suitable for inclusion. The following exclusion criteria were employed to ensure the reliability and validity of the results: (1) Research that excluded essential facts, and (2) Publications without full-text access. 

Data extraction and risk of bias assessment

The following data were extracted: author’s name, year of publication, participant's characteristics, treatment regimens, and the result of the study. Subsequently, we extracted data from our selected articles. Articles were also assessed in terms of quality by using the Consolidated Standards of Reporting Trials (CONSORT) guidelines. Quality assessment was done collaboratively by all reviewers until consensus was reached, as it is shown in Table 1. 

**Table 1:** Characteristics and results of the included studies ESWT: extracorporeal shockwave therapy

Author, year	Population	Age	Intervention	Total sample	Healing rate
Schaden W et al., 2001 [23]	Nonunion or delayed femur fracture	40.6	1 session of ESWT (0.4 mJ/mm^2^, 28 kV, 12000 impulses)	115	91.67%
Xu ZH et al., 2009 [28]	Nonunion femur fracture	38.1	1 session of ESWT (0.62mJ/mm^2^, 28kV, 6000-10000 shock impulses)	22	75.80%
Kuo et al., 2015 [27]	Nonunion femur fracture	30	1 session of ESWT (0.58 mJ/mm^2^, 28 kV, 3000 impulses)	22	63.60%
Sandoval et al., 2017 [27]	Nonunion femur fracture	36.1	3 sessions of ESWT (0.55 mJ/mm^2^, 10000 impulses)	25	52%
Chooi et al., 2004 [27]	Nonunion femur fracture	29.5	1 session of ESWT (25kV, 4000 impulses)	4	75%
Stojadinovic et al., 2011 [27]	Nonunion femur fracture	48	1 session of ESWT	70	72.90%
Rompe et al., 2001 [27]	Nonunion femur fracture	39.5	1 session if ESWT (0.6 mJ/mm^2^, 3000 impulses)	15	80%

There are around 10 cases of femoral shaft fractures per 100,000 persons each year, and intramedullary nail fixation is now the treatment of choice for these injuries [29,30]. Exchange nailing, dynamization of the nail, synthesis with plate and screws, and external fixation are among the therapies that may be administered to patients with pseudarthrosis, which occurs in between 1.9% and 5% of these cases [31]. To aid in the repair of the nonunion focus, any of these procedures might be accompanied by growth factors, autologous or bank bone grafting, or a combination of the two [32].

Determining the type of pseudoarthrosis is crucial because, in the case of hypertrophic pseudoarthrosis, the pathogenesis is related to the poor stability of the fracture site; therefore, treatments that give the fracture more stability will be preferred (e.g., plate and screws or replacing the nail with a larger one). In the case of oligotrophic or atrophic pseudoarthrosis, on the other hand, the pathogenesis will be linked to a likely decrease in the biological stimuli for the healing process, so it will be appropriate to intervene with procedures that encourage the reactivation of the normal reparative processes (e.g., revision of the intramedullary nail with reaming of the intramedullary canal; plate and screws associated with bone graft implantation) [33].

Our systematic review of evidence involving 273 cases resulted in an average healing rate of 73% for ESWT in treating nonunion femur fractures. A systematic review published by Sansone et al. in 2022 reported a healing rate for nonunions of the metatarsals of 90%, followed by the tibia at 75.54%, the femur at 66.91%, and the humerus at 63.64%. Two explanations may account for the different success rates [27]. One possible explanation for the first is that SWs are attenuated before they reach bones by the soft tissues that surround them. Because the soft tissue layer that SWs must pass through is thinner on more superficial bones, such as metatarsals, they may absorb a greater amount of energy. A second justification stems from the fact that, according to the Hopkins effect, a portion of a SW is reflected whenever it moves from a lower-density medium to one with a higher density [27]. Some SWs, after passing through the cancellous bone and the first layer of cortical bone, are reflected off the cortical bone on the other side. In rare instances, these reflected waves may meet antithetical waves, which can increase their intensity. The smaller cortical bone and shorter distance between the two cortical surfaces of metatarsal bones make this effect more likely to apply [34]. In addition, the midfoot and tibia are more amenable to immobilization, which can aid in healing, than the femur and, in particular, the humerus.

SWs, as they travel through a human body, can promote healing and regeneration by creating a variety of mechanical stress effects at the interfaces of different tissues [35]. Physical, chemical, and biological impacts are all conceivable with ESWT, according to the available information [36]. The first thing to know is that SWs create positive and negative pressure on a physical basis. A second SW or fluid microjets are created when air bubbles are generated and imploded at the interfaces of tissues due to cavitation caused by negative pressure, while energy is absorbed, reflected, refracted, and transmitted to the tissues by means of positive pressure [36,37]. Cellular membrane permeability and ionization of biological molecules are both improved by cavitation [38]. On a molecular level, SWs can control cellular transmembrane ion channels, which in turn mediate intracellular calcium flux [39]. Changes in the transmembrane current caused by an increase in Ca2+ inflow can transport data about SW impulses from the extracellular space to the cell nucleus [40]. Thirdly, on a molecular level, SWs have been discovered to have several biological functions, such as enhancing angiogenesis, speeding wound healing, promoting bone non-union repair, nerve regeneration, and suppressing inflammation [41].

It is possible that ESWT-mediated enhancement of fracture healing operates via a variety of pathways. One benefit of ESWT is that it can stimulate the body's osteogenic response and reactivate bone development through the creation of fracture hematomas caused by microfractures [37,42,43]. Secondly, research has shown that ESWT can improve osteoblast differentiation, maintain MSCs' osteogenic capacity, and stimulate osteoblast formation [44,45]. Additionally, ESWT has the potential to inhibit osteoclast formation and development in vitro by focusing on the NF-κB signaling pathway [46]. Thus, it is believed that ESWT facilitates fracture healing by controlling osteoclasts and osteoblasts.

After retrospective research by Valchanou and Michailov suggested using high-energy ESWT on nonunion fractures, the rate of bone union was 85.4% (70 out of 82 patients) [47]. A prospective, nonrandomized research study by Vulpiani et al. that included 143 patients with nonunions found that 61.3% of trophic nonunions (hypertrophic and oligotrophic) healed, but 29.2% of atrophic nonunions did not [48]. While the healing rate varied between 41% and 85%, a comprehensive review conducted in 2011 by Zelle et al. found that hypertrophic nonunions had a union rate of 76% and atrophic nonunions had a rate of 29% [49]. The treatment of atrophying nonunion is complicated since the condition is marked by a lack of callus on radiographs as a result of inadequate biology and vascularization. Several surgical methods have been suggested to treat atrophic nonunion, with the goals of improving mechanical and biological stability. Autologous bone grafting, demineralized bone matrix, bone morphogenetic protein, and parathyroid hormone treatment are some of the methods that aim to restore the biological milieu. Several proangiogenic and pro-osteogenic growth factors can be up-regulated and expressed by ESWT [50,51]. Bony union might occur as a result of this ability's modification of the nonunion environment [52]. Atrophic nonunions have been found to increase the likelihood of bony union (odds ratio 0.23), contributing to the 10% reported failure rate of surgical therapy for long bone nonunions [53]. On the other hand, the already unstable blood flow associated with lengthy bone nonunions may be made worse by any surgical procedure. Atrophic nonunion and the total number of surgeries were found to be independent negative factors that impacted the success of ESWT in treating long bone nonunions, even though ESWT has been shown in animal studies to upregulate and express a variety of proangiogenic and pro-osteogenic growth factors [52].

A major obstacle in fracture fixation therapy is the existence of bone imperfections. According to the available data, critical-sized bone defects are defined as those that measure more than 1-2 cm in diameter and cause a loss of more than 50% of the bone's circumference [54,55]. A continuous fracture gap of varied diameters characterizes fracture nonunion, which is caused by the absence of bone bridging between pieces. Treatment of fracture nonunions with high-energy ESWT has shown efficacy in closing these gaps [56]. While ESWT was successful in achieving bone union in 27 patients with nonunion, four of those patients had a significant fracture gap (>2 cm) on radiographs taken before treatment, according to Vulpiani et al. [48]. Moya et al. found that a nonunion gap larger than 5 mm was associated with worse results after ESWT [56].

## Conclusions

This case report underscores the efficacy of ESWT as a plausible alternative to surgical intervention for nonunion fractures of the femur, especially when conventional treatments have proven ineffective in promoting bone repair. This study's thorough literature evaluation and methodical analysis highlight the effectiveness of ESWT in facilitating bone union, as demonstrated by the successful treatment of the case described. Despite the variety in success rates across different nonunion types, ESWT has shown a notable potential to augment osteogenic responses, accelerate osteoblast differentiation, and promote bone repair via diverse biological mechanisms. This non-invasive method is a favorable alternative for patients with nonunion fractures, minimizing the necessity for further surgical interventions and related complications. Further research is essential to create standardized protocols and enhance treatment parameters for ESWT, ensuring consistent and dependable outcomes across various patient groups.
